# Causal impact of genetically-determined fish and fish oil intake on epigenetic age acceleration and related serum markers

**DOI:** 10.1186/s40246-025-00756-3

**Published:** 2025-05-24

**Authors:** Paul Fabian, Gil Blander, Renee Deehan, Ali Torkamani, Bartek Nogal

**Affiliations:** 1 InsideTracker, One Broadway, 14 th 10 Fl, Cambridge, MA 02142 USA; 2 The Scripps Translational Science Institute, The Scripps Research Institute, La Jolla, CA 92037 USA; 3 Department of Integrative Structural and Computational Biology, The Scripps Research Institute, La Jolla, CA 92037 USA

**Keywords:** Oily fish intake, Two sample Mendelian randomization, 2SMR, Epigenetic aging, Biological aging, Serum biomarkers, Healthspan, Fish oil

## Abstract

**Background:**

The interplay between diet and healthspan is a topic of great interest in biomedical research. Toward this end, consumption of marine omega-3 fatty acids is of particular significance, as reports suggest that diets focused on seafood can prolong the disease-free portion of the human lifespan. Fish consumption has also been linked to reduced biological aging as measured by epigenetic clocks, but there is no conclusive evidence of a causal relationship. Moreover, fish oils reduce triglycerides, and may affect other lipid profiles, as well as systemic inflammation. To investigate further, we used two-sample Mendelian randomization to investigate potential causality between fish intake and healthspan markers.

**Methods:**

Bidirectional Mendelian randomization was performed in the two-sample setting with publicly available GWAS summary statistics. GWAS data from the UK Biobank for oily fish consumption (*n* = 460,443) and fish oil supplementation (*n* = 461,384) were used as the primary exposures. First-generation epigenetic clocks Hannum age and intrinsic epigenetic age acceleration (IEAA), as well as second-generation clocks GrimAge and PhenoAge were collected from an independent dataset of individuals of European ancestry (*n* = [34,449–34,667]). Finally, data from the Integrative Epidemiology Unit database was used for serum proxies of lipidemia and systemic inflammation (*n* = [61,308–78,700]). Additional sensitivity analyses, such as reverse causation testing and the Cochran’s Q test were performed for exposure-outcome pairs where the inverse variance weighted (IVW) method was significant (*p*-value < 0.05), and where the MR Egger method indicated an effect in the same direction as the IVW result.

**Results:**

We report that oily fish consumption appears to decrease PhenoAge acceleration (*p* < 0.0086), whereas fish oil supplementation appears to decrease GrimAge (*p* $=$ 0.037). Both omega-3 exposures modify the epigenetic clocks in the expected negative, or age-decelerating, direction. For the serum biomarkers, we find evidence that fish oil consumption leads to a reduction in triglycerides (*p* $=$ 0.004), although HDL and LDL were not significantly modified. Finally, we also detected a suggestive inverse relationship between oily fish consumption and hsCRP (*p* $=$ 0.064).

**Conclusions:**

Our analysis shows that consuming fish oil, whether through whole food or as a supplement, can have a rejuvenating impact as measured by PhenoAge and GrimAge acceleration. We have also provided evidence further linking fish oil intake and lower triglyceride levels. These results, based on robust MR-based analyses, emphasize the effectiveness of dietary choices in modifying emerging measures of healthspan.

## Background

The nexus between diet and health outcomes is a topic of enduring interest in biomedical research. Among a myriad of dietary components, the consumption of fatty fish and/or supplementation with fish oils has gained attention due primarily to the associated dietary enrichment in omega-3 polyunsaturated fatty acids (PUFAs), as well as vitamin D, selenium, and antioxidants such as astaxanthin [1–4].

Furthermore, multiple reports have suggested that wholefood diets focused on seafood in lieu of meat consumption as the protein source are beneficial toward extending healthspan, or the disease-free portion of an individual’s lifespan [5, 6]. While no universally accepted measure of healthspan exists, multiple ’biological clock’ estimators have been developed toward helping to identify lifestyle and other interventions that may promote healthspan extension [7–11]. Some of the most well characterized of these are the DNA methylation-based clocks, two of which have now been developed over the past decade and all of which have been shown to be associated with multiple health outcomes, beyond the limitations of chronological age [8–12]. These epigenetic clocks include the first-generation Horvath and Hannum clocks, and the second-generation PhenoAge and GrimAge clocks, and they appear to offer intriguing aggregate readouts of the effects of dietary and/or lifestyle factors on human healthspan [8–11].

Of particular interest are the GrimAge and PhenoAge epigenetic clocks, both of which integrate additional clinical chemistry biomarkers along with chronological age to create a new predictive measure towards examining how mortality risk differs in a population of the same chronological age [9]. In the case of PhenoAge, a new measure of ’phenotypic age’ was constructed such that alongside chronological age, 9 biomarkers that correlated with mortality risk were factored into the model. By strengthening the signal of phenotypes associated with aging-related diseases in the predictive outcome, PhenoAge served as a more relevant measure of biological aging than chronological age alone, and therefore uncovered novel CpG sites associated with changes in aging-related phenotypes.

GrimAge, the more recent of the second-generation clocks, was constructed by training separate DNA Methylation (DNAm) models on smoking pack-years, and other plasma proteins such as C-reactive protein, and adrenomedullin. All of these DNAm-biomarker proxies in combination with demographics such as chronological age and sex were then regressed on time-to-death data to create the DNAmGrimAge metric [8].

Both of these epigenetic clocks have unique strengths in terms of their predictive ability. DNAmGrimAge strongly predicts time-to-death, and time to incidence of coronary artery disease (CAD) and multiple cancer types, while PhenoAge consistently performs well in predicting all-cause mortality, cancers, healthspan traits, and Alzheimer’s disease [8, 9]. Mechanistic insights are an active area of investigation, with potential focus areas for both DNAmPhenoAge and DNAmGrimAge including inflammatory responses, immune function, metabolic regulation, and broader aspects of cellular health [8, 9].

Toward this end, there is some evidence for omega-3 PUFAs such as those comprising fish oils associating with methylation patterns that suggest decelerated aging [13–15]. For example, one cross-sectional study examining the effects of lifestyle factors on the first-generation epigenetic clocks found that fish consumption had a significant, albeit weak, decelerating effect on epigenetic aging as measured by the immune system responsive Hannum clock [16]. However, due to the self-reported nature of the dietary habits and the assumption of non-confounding in this observational study, no causal inference could be established [16].

Further evidence is emerging regarding the modification of PhenoAge via Omega 3 intake and other lifestyle factors. A recent cross-sectional study of over 20, 000 individuals from the NHANES cohort examined the impact of Omega-3 fatty acids on phenotypic age acceleration (PhenoAge). The study found a significant negative correlation between Omega-3 intake and PhenoAge acceleration (*p* $= 0.004$). A dose-response effect was also detected, and it was reported that 1.103 gs per day was sufficient to moderate PhenoAge acceleration, with an attenuated impact for doses beyond this threshold [17].

Zhao et al. examined the interplay between lifestyle, PhenoAge, and mortality risk, finding that adherence to the life’s essential 8 (LE8) recommendations-such as maintaining a nutritious diet, engaging in regular physical activity, and controlling cholesterol-helped slow phenotypic aging measured by the PhenoAge metric. Highlighting PhenoAge’s responsiveness to lifestyle, they showed PhenoAge mediated 36 percent of LE8’s effect on all-cause mortality [18].

Similarly, Carbonneau et al. found that better adherence to the LE8 was associated with slower GrimAge acceleration, underscoring the role of GrimAge as a readout of lifestyle influences on cardiovascular disease and mortality [19]. These findings are further supported by a study that examined the effects of vitamin D, omega-3, and exercise on DNA methylation, as measured by second and third generation epigenetic clocks. The study analyzed each factor individually and in aggregate, and found that Omega-3 slowed aging for PhenoAge, GrimAge2, and DunedinPACE, with a further dose effect of all three factors on PhenoAge [20].

In addition to the emerging measures of healthspan, reports also suggest that fish oils exert beneficial health effects via the modulation of lipid profiles, primarily through triglycerides (Tg), but also through modification of high- and low-density lipoproteins (HDL and LDL, respectively), though results for the latter are less consistent [21–27]. Furthermore, there is some evidence that fish oil may lower systemic inflammation as measured by high-sensitivity C-reactive protein (hsCRP), another significant proxy for cardiovascular disease and healthy aging [28–30].

The above-mentioned intervention effects of fish oils on epigenetic and traditional biomarkers of healthspan are compelling, though to our knowledge, there have not been investigations that would enable causal inference with regards to fatty fish consumption and/or fish oil supplementation and healthspan extension as measured by the emerging biological clocks, such as epigenetic age acceleration. With this in view, two-sample Mendelian randomization (2SMR) is a method that uses fixed genetic instrumental variables to infer causal relationships from observational data by leveraging genome-wide association study (GWAS) summary statistics [31]. By employing genetic variants as instruments, 2SMR mimics a randomized controlled trial design, providing more robust evidence for causality, particularly due to the large sample sizes used. Thus, to investigate a causal relationship between marine omega-3 intake and its effects on measurable proxies of healthspan, we performed 2SMR analyses with fish oil exposures and the above mentioned epigenetic and serum biomarkers of healthspan.

## Results

### Impact of oily fish intake on epigenetic age acceleration

**Fig. 1:**
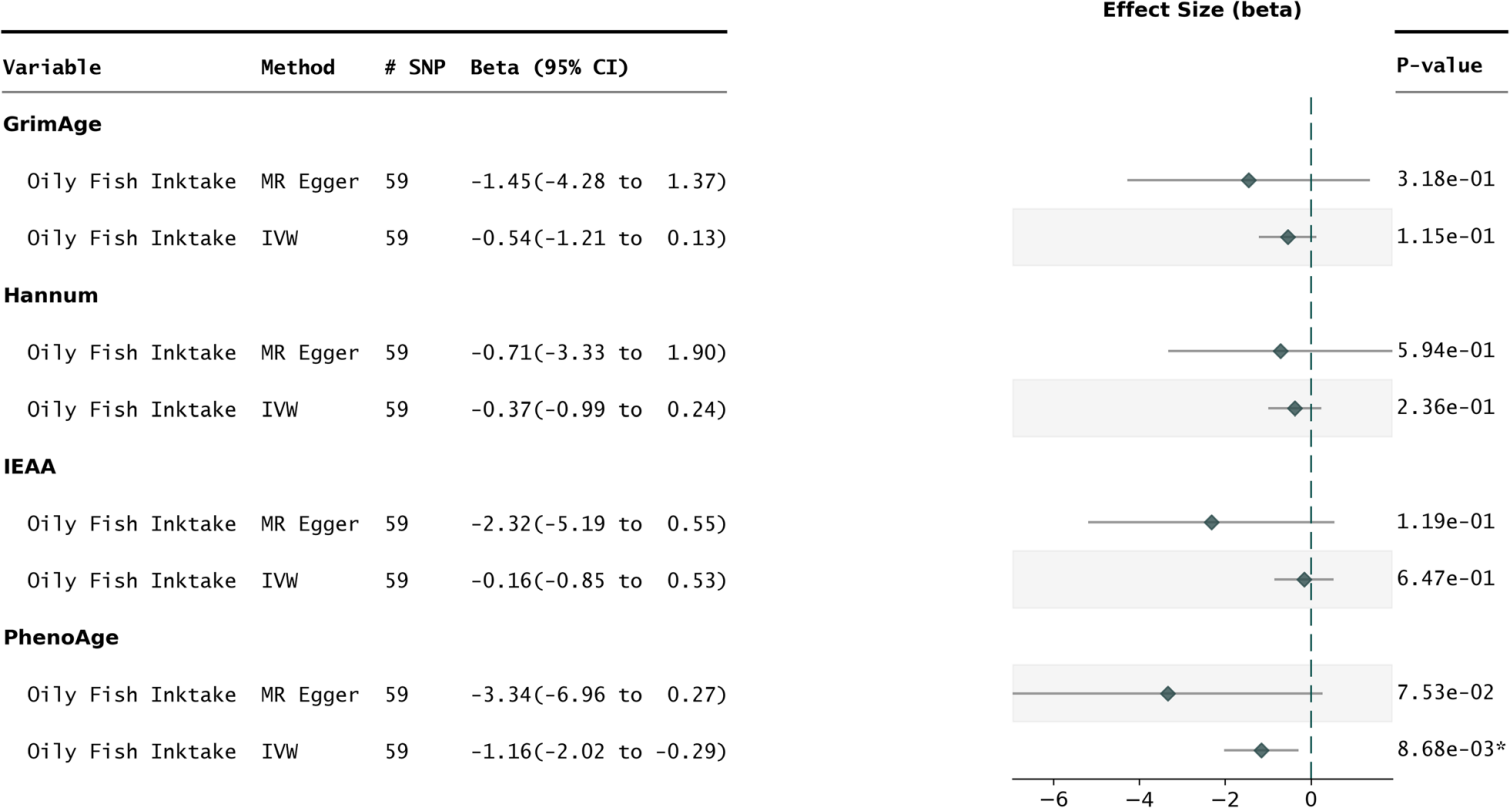
Oily Fish intake vs epigenetic aging clocks. Forest plot representing the results of 2SMR performed with oily fish intake as the exposure vs first- and second-generation epigenetic clocks. The IVW and MR Egger methods are shown for each exposure outcome pair

With oily fish intake as the exposure, 59 independent single nucleotide polymorphisms (SNPs) were identified as instrumental variables (IVs) (Table 1) for use against each of the four biological clock outcome datasets. 2SMR performed for each of these exposure outcome pairs yielded a directionally consistent effect for the IVW method on all four epigenetic age acceleration measures (Fig. 1). Of these four, only PhenoAge reached statistical significance (PhenoAge $P_{IVW} = 0.0086$).

**Table 1:** Characteristics of genetic variants used as instrumental variables in the oily fish intake and fish oil supplementation exposures

SNP	Corresponding gene	Consequence	AF
Fish oil supplement			
rs4861024	BEND4	Intron variant	0.3379
rs2533273	DPP6	Intron variant	0.4046
rs3789045	LRRN2	3 Prime Utr variant	0.1843
rs9870832	-	Intergenic variant	-
Oily fish intake			
rs13070166	CADM2	Intron variant	0.2484
rs9886779	CCDC171	Intron variant	0.4748
rs11859365	CDH13	Intron variant	0.2776
rs7683782	CPEB2-DT	Intron variant,non coding transcript variant	-
rs61882686	DGKZ	Intron variant	0.0347
rs2374424	DLG2	Intron variant	0.5891
rs12896749	EML1	Intron variant	-
rs11767283	FEZF1	Upstream gene variant	0.2905
rs1876245	FOXP1	Intron variant	-
rs1421085	FTO	Intron variant	0.2286
rs9301837	GPC5	Intron variant	0.3718
rs6059844	ITCH	Intron variant	0.4407
rs4510068	KANSL1	Intron variant	-
rs790564	LINC01414	Intron variant,non coding transcript variant	0.6901
rs17050031	LINC01793	Intron variant,non coding transcript variant	-
rs510161	LINC02742	Intron variant,non coding transcript variant	0.2666
rs10076975	LINC03000	Intron variant,non coding transcript variant	-
rs45501495	LRRN2	Intron variant	-
rs55985303	LRRTM4	Intron variant	0.4728
rs4002471	MAMSTR	Downstream Gene Variant	0.2300
rs6089753	MIR1-1HG	Intron Variant,Non Coding Transcript Variant	0.4435
rs10828250	MLLT10	Intron variant	0.1388
rs11986122	MSRA	Intron variant	-
rs973526	NEGR1	Intron variant	0.5302
rs12983532	PGPEP1	Intron variant	0.3151
rs10510554	RARB	Intron variant	0.4716
rs303817	SCN8 A	Intron variant	0.5038
rs28533540	SEMA6D	Intron variant	0.4169
rs9841174	SERPINI2	Missense variant	-
rs6465487	SLC25 A13	Intron Variant	0.4669
rs4982738	SLC7 A8	Intron Variant	0.4305
rs12663865	SMIM8	Intron variant,nmd transcript variant	0.7194
rs1201289	TBL1XR1	Intron variant	0.4617
rs10061973	TENM2	Intron variant	-
rs275160	TEX41	Intron variant,non coding transcript variant	-
rs12855717	TMTC4	Downstream Gene Variant	-
rs10513136	ZBTB38	Intron variant	0.0258
rs703987	ZMIZ1	Intron variant	-
rs75887709	ZNF568	Intron variant	0.0535
rs9606833	-	Intergenic variant	0.1198
rs114497213	-	Intron variant,non coding transcript variant	0.0262
rs6033437	-	Intergenic variant	0.4147
rs2827161	-	Intron variant,non coding transcript variant	-
rs55930451	-	Intron variant,non coding transcript variant	0.1725
rs16891727	-	Intergenic variant	0.1072
rs4869859	-	Intergenic variant	0.6292
rs905575	-	Intron variant,non coding transcript variant	-
rs7254235	-	Intron variant,non coding transcript variant	0.5156
rs4278546	-	Intergenic variant	0.5875
rs631490	-	Intergenic variant	-
rs35287743	-	Intergenic variant	-
rs1361016	-	Intron variant,non coding transcript variant	0.8437
rs3124402	-	Intergenic variant	0.5952
rs9597870	-	Intergenic variant	0.1733
rs1951286	-	Intron variant,non coding transcript variant	-
rs59355765	-	Intron variant,non coding transcript variant	0.0949
rs7243428	-	Intron variant,non coding transcript variant	0.1605
rs9958909	-	Intergenic variant	-
rs9958909	-	Intron variant,non coding transcript variant	-
rs28623270	-	Upstream gene variant	0.2532
rs9889161	-	Intron variant,non coding transcript variant	0.2907
rs552234	-	Intergenic variant	0.4010

**Fig. 2:**
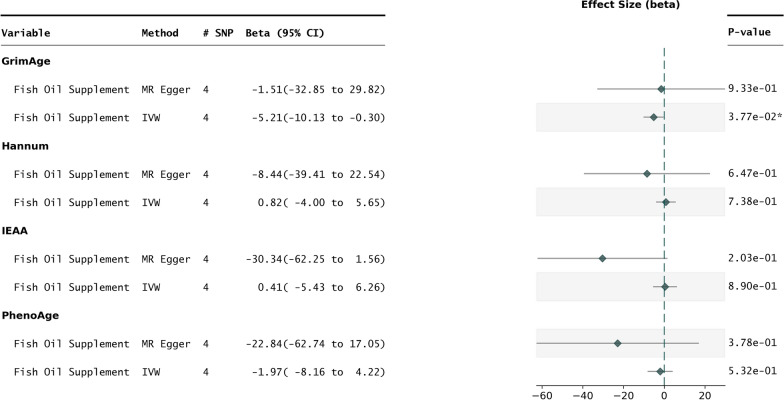
Fish oil vs epigenetic aging clocks. Forest plot representing the results of 2SMR performed with fish oil supplementation as the exposure vs first- and second-generation epigenetic clocks. The IVW and MR Egger methods are shown for each exposure outcome pair

To further scrutinize the relationship between Oily Fish Intake and PhenoAge, additional MR methods including the simple mode, weighted mode, and weighted median were employed. These three methods also yielded a directionally consistent effect with respect to the IVW method, towards the age-decelerating direction. (Figure 1 S). Further sensitivity tests included the leave-one-out analysis to test for the overpowering influence of any single genetic instrument on the overall estimate, and the MR Egger Intercept to test for horizontal pleiotropy (See heterogeneity p-value in Table 2), to ensure no significant heterogeneity across instruments. In each test, there were no significant confounding effects that influenced this relation (Figure 2 S).

**Table 2:** Statistically significant results across all exposure outcome pairs. All 5 MR methods are shown

Exposure	Outcome	Method	N_SNP	b	se	*p*-value	Heterogeneity *p*-value
Oily Fish Inktake	PhenoAge	MR Egger	59	$-$3.34	1.84	7.53e-02	0.23
Oily Fish Inktake	PhenoAge	Weighted median	59	$-$1.00	0.62	1.10e-01	0.23
Oily Fish Inktake	PhenoAge	IVW	59	$-$1.16	0.44	8.68e-03*	0.23
Oily Fish Inktake	PhenoAge	Simple mode	59	$-$0.90	1.46	5.41e-01	0.23
Oily Fish Inktake	PhenoAge	Weighted mode	59	$-$0.90	1.37	5.16e-01	0.23
Fish Oil Supplement	GrimAge	MR Egger	4	$-$1.51	15.99	9.33e-01	0.84
Fish Oil Supplement	GrimAge	Weighted median	4	$-$4.51	2.88	1.17e-01	0.84
Fish Oil Supplement	GrimAge	IVW	4	$-$5.21	2.51	3.77e-02*	0.84
Fish Oil Supplement	GrimAge	Simple mode	4	$-$3.88	3.93	3.96e-01	0.84
Fish Oil Supplement	GrimAge	Weighted mode	4	$-$3.80	3.87	3.98e-01	0.84
Fish Oil Supplement	Tryglycerides	MR Egger	4	$-$0.09	2.59	9.76e-01	0.72
Fish Oil Supplement	Tryglycerides	Weighted median	4	$-$0.93	0.47	4.85e-02*	0.72
Fish Oil Supplement	Tryglycerides	IVW	4	$-$1.15	0.40	4.00e-03*	0.72
Fish Oil Supplement	Tryglycerides	Simple mode	4	$-$0.88	0.67	2.82e-01	0.72
Fish Oil Supplement	Tryglycerides	Weighted mode	4	$-$0.82	0.63	2.83e-01	0.72

**Fig. 3:**
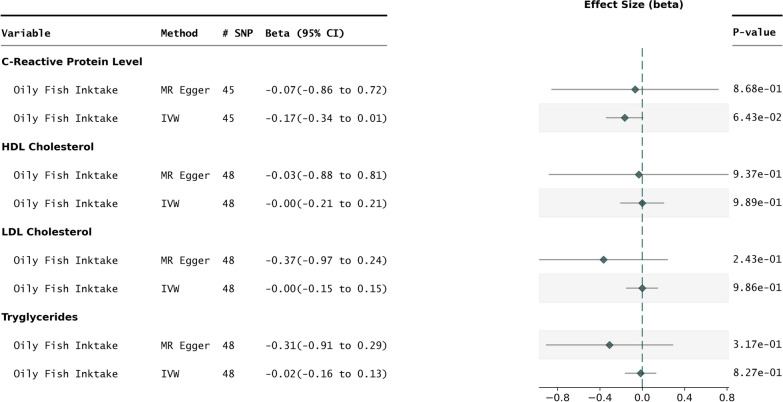
Oily fish intake vs biomarkers. Forest plot representing the results of 2SMR performed with oily fish intake as the exposure vs select lipid and inflammatory biomarkers. The IVW and MR Egger methods are shown for each exposure outcome pair

**Fig. 4:**
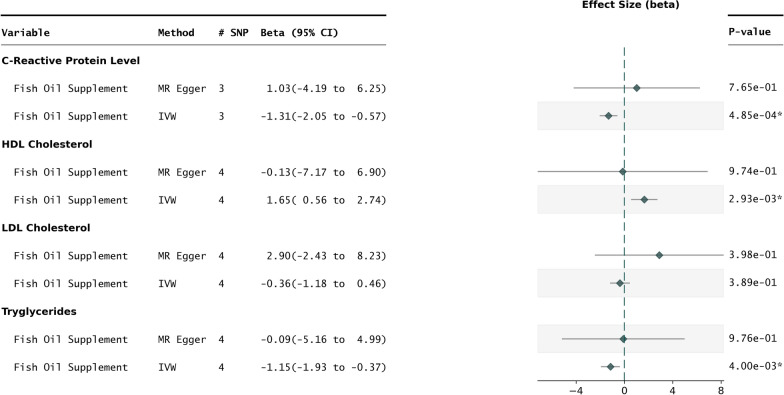
Oily fish intake vs biomarkers. Forest plot representing the results of 2SMR performed with fish oil consumption as the exposure vs select lipid and inflammatory biomarkers. The IVW and MR Egger methods are shown for each exposure outcome pair. For C-Reactive protein and HDL cholesterol, the MR Egger and IVW methods are not directionally consistent, indicating potential pleiotropy

**Fig. 5:**
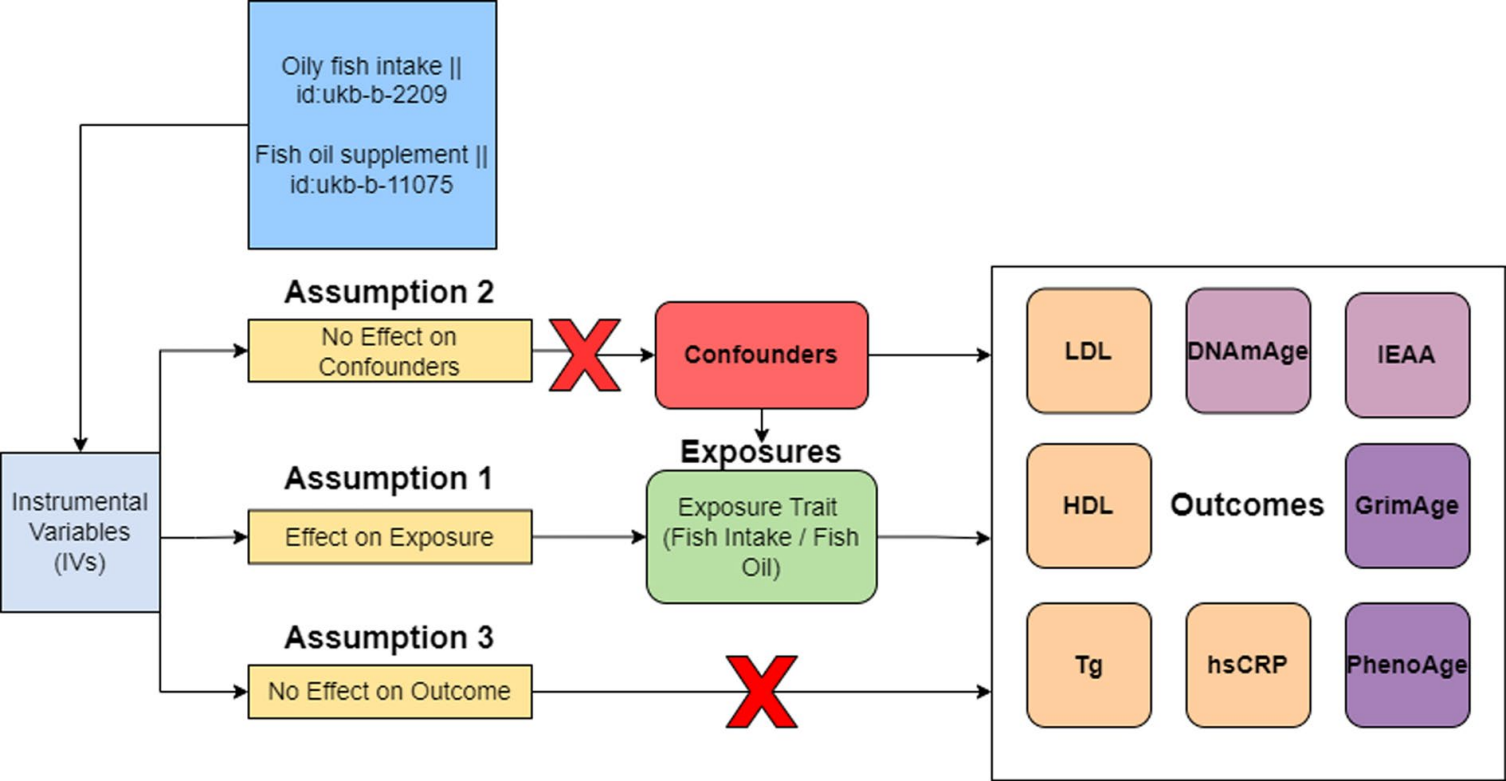
Core assumptions underpinning 2SMR analysis. Three primary assumptions include: 1. Effect of IV on Exposure. 2. No confounding effect on exposure. 3. No confounding effect on outcome. Violations of any of these assumptions can lead to biased MR estimates

**Fig. 6:**
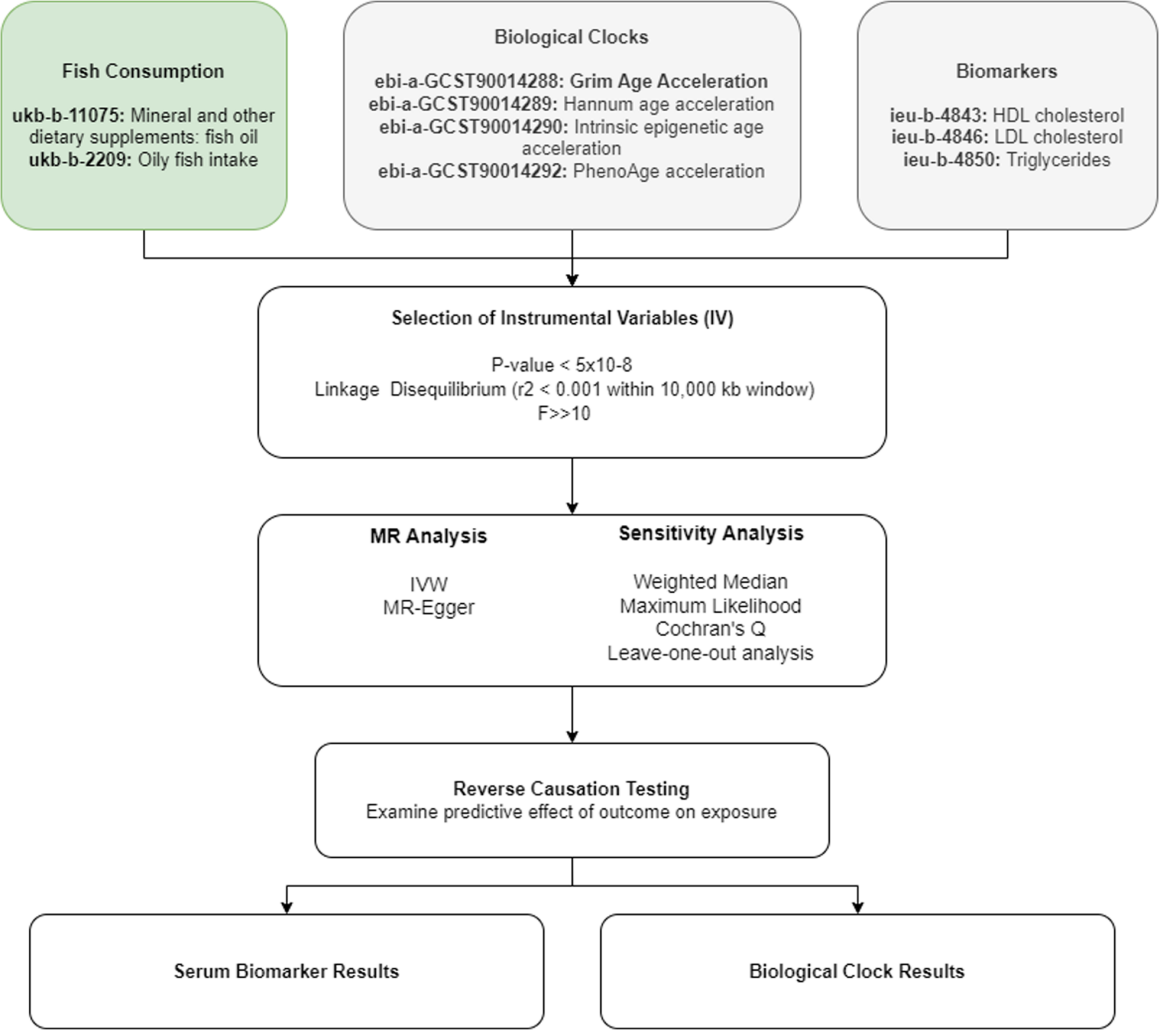
MR methods Overview of the Mendelian randomization workflow assessing the impact of fish consumption on epigenetic aging and biomarkers. Genetic instruments meeting established thresholds for significance and linkage disequilibrium were selected, outlier variants were removed, and multiple MR analyses were performed, followed by reverse causation testing

### Impact of Fish oil supplementation on epigenetic age acceleration

For fish oil supplementation, 4 independent SNPs were used as IVs against all four biological clock outcomes (Table 1). In this case, PhenoAge was not significantly modified (PhenoAge $P_{IVW} > 0.50$) by the exposure, though it remained directionally consistent towards age deceleration. GrimAge, however, did reach statistical significance (GrimAge $P_{IVW} = 0.037$), with the MR-Egger method also showing directional consistency (Fig. 2).

For this fish oil and GrimAge exposure outcome pair, all five MR methods yielded directional consistency (Figure 3 S). The leave-one-out analysis did not reveal any individual SNP that overinfluenced the result, and the heterogeneity p-value was less than 0.1, indicating confounding from horizontal pleiotropy is not significant (Figure 4 S).

### Impact of oily fish intake on select serum biomarkers

To ascertain the established effects of oily fish intake on certain serum lipidemia and inflammatory biomarkers, 2SMR was performed on each exposure outcome pair. There were no statistically significant results between oily fish consumption and the evaluated serum lipid biomarkers, although in the case of C-reactive protein, the result approached statistical significance ($P_{IVW} = 0.064$), with both the IVW and MR Egger methods showing directional consistency (Fig. 3).

### Impact of fish oil supplementation on select serum biomarkers

In the case of fish oil supplementation, there was a statistically significant relationship towards modifying triglycerides in the expected negative direction ($P_{IVW} = 0.004$), with MR-Egger showing directional consistency (Fig. 4). The fish oil supplementation exposure also produced a significant interaction with hsCRP and HDL via the IVW method ($P_{IVW} < 0.01$), however in each case the estimated effect of the MR Egger method was not directionally consistent, indicating potential confounding by pleiotropy (Figure 5 S).

Further analysis was performed for the fish oil and triglyceride exposure outcome pair. The directional effect of the weighted mode, weighted median, and simple mode MR methods all aligned with the IVW method towards triglyceride reduction. As in the prior comparisons, all of the sensitivity tests including the leave-one-out analysis and Cochran’s Q test did not indicate significant confounding (Figure 6 S, Table 2).

### Reverse causation testing

To further validate our results, reverse causation analysis was performed to explore whether there was a significant effect of the outcome on the exposure. In each case (Oily Fish and PhenoAge, Fish Oil and GrimAge, Fish Oil and Triglycerides), there was no evidence for reverse causality, as shown in the following figures (Figure 7 S, 8 S, 9 S, 10 S). All statistically significant results that passed the sensitivity tests are presented in Table 2.

## Discussion

### Overview

In this 2-sample MR study, we attempt to augment the understanding of the beneficial effects of oily fish consumption on certain measures of healthy aging. Toward this end, we provide evidence for potential causal effects of oily fish and fish oil intake on certain emerging epigenetic measures of biological age. Whole oily fish consumption showed a potential causal relationship with PhenoAge acceleration, whereas supplemental fish oil had evidence for a potential causal relationship with GrimAge acceleration. We also report an overall directionally consistent effect of oily fish consumption on first- and second-generation epigenetic age acceleration measures. In addition, we find supportive evidence for potential causality between fish oil supplementation and triglyceride reduction, though not HDL or LDL, suggesting that this intervention is unlikely to directly modify serum cholesterol levels.

The first and second generation epigenetic clocks estimate biological age using DNA methylation patterns. The former, namely, the Horvath and Hannum clocks, predict chronological age across cells and tissues, but were not originally trained to account for age-related changes in physiological function [10, 11]. However, second-generation clocks, such as PhenoAge and GrimAge, capture intervention effects related to healthspan, physiological function, and mortality risk. This key distinction between the first- and second-generation epigenetic clocks may explain why the dietary interventions examined in the current study exhibited a significant modulating effect only on the latter.

### PhenoAge and GrimAge response to lifestyle and dietary factors

Both PhenoAge and GrimAge were trained on serum biomarkers related to healthspan, such as hsCRP [8, 9, 32]. Indeed, a recent investigation reported that higher diet quality, as measured by the Dietary Approaches to Stop Hypertension (DASH) score, was associated with decelerated epigenetic aging only in the second (and third) generation epigenetic clocks, including GrimAge (p < 0.001) and PhenoAge (*p* $=$ 0.001) [33]. Hence, it is the latter generation clocks that have proven to be more responsive to dietary and/or other lifestyle interventions [16, 34–36].

In this analysis, we do observe that PhenoAge and GrimAge acceleration are significantly modified in the direction of rejuvenation for either whole food-based and/or supplemental exposures to fish oils. This result appears to be in line with ample evidence that points to the general cardio-protective effects of omega-3 PUFA [37–39], which is in alignment with reports that CVD accelerates aging as measured by the two second-generation epigenetic clocks [40].

### Fish oil consumption and serum biomarkers

Multiple reports suggest that oily fish and/or supplemental fish oil intake impart health benefits via improved cardiovascular health and systemic inflammation levels, though, aside from its effects on triglycerides, the effects of this intervention on serum lipid and inflammatory markers appear to be less convincing [41–43]. Systematic reviews that assessed the impact of fish or fish oil consumption on vascular risk factors found that it was associated with a reduction in plasma triglyceride levels, as well as an increase in HDL cholesterol levels [25, 44]. Another meta-analysis of 191,558 individuals reported that higher fish intake was linked to lower triglyceride levels, however no associations were found with serum HDL, whereas LDL exhibited a somewhat paradoxical increasing trend with increased consumption of fish [41].

With respect to the effects of oily fish intake on systemic inflammation, the ATTICA study reported that individuals who consumed more than 300 g of whole fish per week had significantly lower levels of all measured inflammatory markers including hsCRP, while supplemental fish oil’s effects on established clinical inflammatory biomarkers such as hsCRP were less consistent [42, 43, 45]. For example, Yang et al. noted a reduction in hsCRP induced by cod liver oil in gestational diabetics, but the effect was null in type 2 diabetics [42, 43].

We note that our 2SMR-based analysis of oily fish intake and fish oil supplementation aligns with the above-mentioned effects of these interventions on serum biomarkers, where fish oil supplementation was found to only causally reduce serum triglycerides. Interestingly, oily fish consumption did not modify this biomarker in our investigation, which may be a property of the wholefood based exposure not supplying enough omega-3 PUFAs to induce an intervention effect. When these fatty acids are consumed in quantities above 3 g daily, a significant decrease in serum triglycerides is observed, with a potential mechanism being a reduction in the production of VLDL-TG in the liver, a key contributor to plasma triglyceride levels [23].

The absence of a similar effect from oily fish consumption in our study might therefore be attributed to wholefood-based exposure not providing sufficient omega-3 PUFAs to elicit this intervention effect. Finally, we did observe a mild hsCRP-reducing effect of oily fish intake, which is consistent with the literature [45] and may be explained by a general anti-inflammatory effect of various components of fish, including the antioxidants selenium and astaxanthin, which have been associated with attenuated levels of this serum and other serum proxies of systemic inflammation [46–49].

### Fish oil consumption genetic variants relate to multiple nutrient-seeking behaviors

Although we selected genetic instruments that are robustly associated with our exposures of interest, which should minimize the probability of a biased causal inference, we wanted to ascertain that the individual genetic variants are indeed related to dietary behaviors. Toward this end, we note that several of the variants within oily fish and fish oil intake exposures contribute to the variance within several nutrient-seeking habits, the most well-characterized of these being rs1421085 within the *FTO* locus. Multiple studies have implicated this and other *FTO* variants in nutrient-seeking behaviors, both directly and via gene-diet interaction [50–52]. For example, rs7206790, which is in linkage disequilibrium (D’ = 0.84) with the genetic instrument used in the current study, was found to be associated with EPA and DHA consumption within the CHARGE consortium at near genome-wide significant level [50]. Moreover, rs1421085 has also previously been indicated in dietary protein-seeking, independent of its effects on adiposity [52].

Additionally, recent UK Biobank analyses have revealed strong interactions of this causal variant with dietary variation [51]. Another variant, rs11859365, lies within the *CDH13* gene, which harbors a quantitative trait locus (QTL) of the metabolically protective adipokine, adiponectin [53]. To this point, rs11859365 has, at genome-wide significant level, been linked to liking and/or consumption of specific foods, including fish, herring, salmon, mackerel, sardines, seafood, pork, capers, and savory foods [54]. Additionally, our PheWAS has revealed that the SNP rs2533273 within the fish oil supplement exposure is also associated with other dietary behaviors, including dried and fresh fruit intake, vitamin supplementation, poultry, pork, tea and alcohol intake. In conjunction with our sensitivity analyses, the above observations provide evidence that fish consumption exposures examined in the context of the current study are unlikely to violate the above-mentioned MR assumptions that must hold to enable causal inference.

### Study limitations

This study has several limitations. To minimize confounding by population stratification, our results are based on European-ancestry populations, and may not generalize to more ethnically diverse populations due to different genetic backgrounds and dietary habits. Although Mendelian randomization (MR) methods offer robust evidence, the assumptions might not always hold, especially with unrecognized pleiotropy or unaccounted pathways. The use of novel second-generation epigenetic age measures may not fully capture all biological aging aspects. Therefore, while our results suggest potential causal relationships, they must be cautiously interpreted, and their clinical relevance is still to be determined.

## Conclusions

In summary, our 2SMR analysis suggests that increased fish oil intake either in the form of whole food or as a supplement may exert a rejuvenating effect as measured by PhenoAge or GrimAge acceleration, respectively. We also provide additional evidence of a potential causal relationship between fish oil consumption and triglyceride reduction. Given these MR-based analyses are unlikely to be confounded, they underscore the efficacy of dietary habits in the modulation of healthspan.

## Methods

### Study design

Drawing causal inferences using 2SMR requires strong adherence to 3 critical assumptions [31]. First, the chosen IVs - in this case, SNPs associated with oily fish intake—must show a robust association with the exposure of interest. Second, the IVs should not be directly associated with the outcome or through any confounding variables. Lastly, the IVs must influence the outcome exclusively through the exposure (Fig. 5). If the 3 above mentioned assumptions hold, MR-estimate effects of exposure on outcomes are not likely to be significantly affected by reverse causation or confounding [55].

### GWAS summary statistics

The exposure datasets for oily fish intake (ukb-b-2209; $N = 460,443$) and fish oil consumption (ukb-b-11075; $N = 461,384$) originate from the MRC-IEU consortium, and are composed of both males and females of European ancestry. These datasets are publicly available, and no ethical approval was required. Only those instruments with genome-wide significance (*p* $< 5e-8$) were used as IVs. GWAS summary statistics from the Integrative Epidemiology Unit (ieu) database were used as the blood biomarker exposures. Specifically HDL cholesterol (id:ieu-b-4843; $N = 37,120$), LDL cholesterol (id:ieu-b-4846 $N =70,814$), triglycerides (id:ieu-b-4850; $N = 78,700$), and C-reactive protein (id:ieu-b-35, $N = 204,402$) from the Within Family GWAS Consortium were used. In each dataset, all, participants were of European descent to minimize population stratification. Additionally, all exposure/outcome datasets had undergone adjustment for principal components to adjust for population structure as described in Bycroft et al [56]. Epigenetic datasets, including GrimAge, Hannum, PhenoAge and intrinsic epigenetic age acceleration (IEAA), were derived from McCartney et al., the largest epigenetic aging GWAS to date, with samples sizes ranging from 34, 449 to 34, 467 individuals [57]. Figure 6 outlines the entire methods process.

### Instrumental variable selection

We pruned the IVs for linkage disequilibrium (LD), which removed SNPs exhibiting genome-wide significance for a given exposure (*p* $< 5e-8$) and fell within a 10, 000*kb* window with a correlation of $r^2 > 0.001$. These exposure-associated SNPs, were then utilized as IVs in the MR analysis. To ensure consistency between alleles across the exposure and outcome datasets, palindromic and ambiguous SNPs were removed from the data. Additionally, the F-statistic was used to confirm the strong correlation of the IV with the exposure, such that IVs with $F < 10$ were removed from the analysis. This value was calculated using the formula below:

1 $$\begin{aligned} F = \left( \frac{R^2/k}{([1-R^2]/[n-k-1])}\right) \end{aligned}$$

where $R^2$ is the proportion of explained variability from the IVs of the exposure dataset, *n* is the sample size, and *k* is the number of IVs.

### MR analysis

The exploration of potential causal relationships for each exposure-outcome pair involved two primary MR methods, including Inverse-variance weighting (IVW) and MR Egger [31, 55]. The multiplicative random-effects IVW method is a weighted regression of instrument-outcome effects on instrument-exposure effects with the intercept is set to zero. This method generates a causal estimate of the exposure trait on the outcome traits by regressing the, for example, SNP-oily fish intake trait association on the SNP-epigenetic age measure association, weighted by the inverse of the SNP-epigenetic age measure association, while constraining the intercept of this regression to zero. However, this constraint can result in unbalanced horizontal pleiotropy whereby the instruments influence the outcome through causal pathways distinct from that through the exposure (which would violate the second assumption described above). Such unbalanced horizontal pleiotropy may not represent a true association between the exposure and the outcome, exaggerating or attenuating the effect estimate from the IVW method. However, unbalanced horizontal pleiotropy can be readily assessed by the MR Egger method (via the MR Egger intercept), which provides a valid MR causal estimate that is adjusted for the presence of such directional pleiotropy, albeit at the cost of statistical efficiency. Consistency across effect sizes from these MR methods was evaluated, and the IVW results were deemed significant with a p-value $< 0.05$.

### Sensitivity analyses

We further assessed pleiotropy explicitly for each exposure–outcome pair, retaining only those results with a pleiotropy p-value $> 0.1$. Leave-one-out was then used to to ensure that no single variant excessively influenced the overall effect estimate. We also conducted a reverse causation test to exclude the possibility that the outcome might drive changes in the exposure. Finally, we applied the MR Steiger test to verify that each instrument explained more variance in the exposure than in the outcome, validating the assumed direction of causality [31].

Furthermore, the variant effect predictor (VEP) was applied to each IV of the oily fish intake dataset to determine the implicated genes, and performed a PheWAS across the IVs to ascertain relationships with nutrient-seeking behaviors [70] (see https://gwas.mrcieu.ac.uk/phewas/#info) [58].

### Statistical analyses

The TwoSampleMR package in R was used for the 2SMR analysis, with additional R software packages being utilized for data formatting (see data availability statement) [59]. For each exposure-outcome pair, the IVW method was considered significant with a *p*-value $< 0.05$.

## Additional file

Supplementary file 1.

Supplementary file 2.

Supplementary file 3.

## Data Availability

The 2S-MR analysis was performed using publicly available datasets via the TwoSampleMR R package.

## Abbreviations

PUFAs: Polyunsaturated Fatty Acids
DNA: Deoxyribonucleic acid
GWAS: Genome-wide association study
IV: Instrumental variable
2SMR: Two sample Mendelian randomization
MR: Mendelian randomization
SNP: Single-nucleotide polymorphism
LDL: Low-density lipoprotein
HDL: High-density lipoprotein
Tg: Triglycerides
LOO: Leave one out analysis
hsCRP: High-sensitivity C-reactive protein
DASH: Dietary approaches to stop hypertension
VEP: Variant effect predictor
CAD: Coronary artery disease
NHANES: National health and nutrition examination survey
LE8: Life’s essential 8
CVD: Cardiovascular disease
QTL: Quantitative trait locus
$P_{IVW}$: Inverse variance weighted *p*-value
PheWAS: Phenome wide association studies

## Code availability

Additional code available upon request.
