# Supplementary material for: Causal impact of genetically-determined fish and fish oil intake on epigenetic age acceleration and related serum markers
Source: Hum Genomics. 2025 May 24;19:61. doi: 10.1186/s40246-025-00756-3 (PMC12102828; doi:10.1186/s40246-025-00756-3)
Supplement: Supplementary file 1 [file 40246_2025_756_MOESM1_ESM.pdf]

8 Supplementary Figures

Variable	Method	# SNP	Beta (95% CI)	P-value
**PhenoAge**				
Oily Fish Inktake	MR Egger	59	-3.34(-6.96 to 0.27)	7.53e-02
Oily Fish Inktake	Weighted median	59	-1.00(-2.22 to 0.23)	1.10e-01
Oily Fish Inktake	IVW	59	-1.16(-2.02 to -0.29)	8.68e-03*
Oily Fish Inktake	Simple mode	59	-0.90(-3.75 to 1.96)	5.41e-01
Oily Fish Inktake	Weighted mode	59	-0.90(-3.59 to 1.79)	5.16e-01

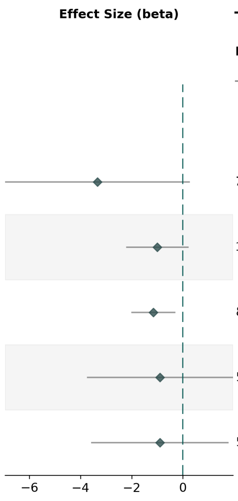

Fig. 1S All 5 MR Methods for Oily Fish Intake vs PhenoAge Forest plot showing additional MR methods of simple mode, weighted mode, and weighted median for this statistically significant exposure outcome pair.

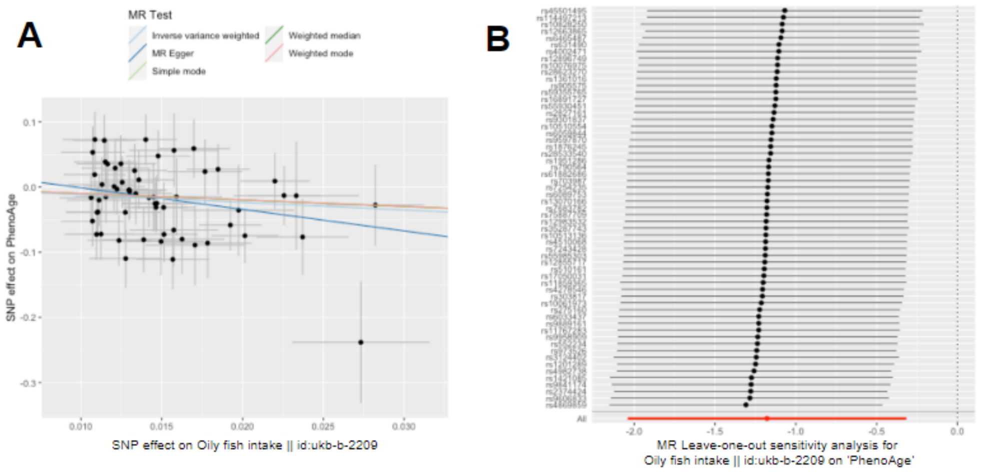

Fig. 2S Sensitivity Analysis of Oily Fish Intake on PhenoAge. Scatter plot of IV effects on 5 MR methods (A) and LOO analysis (B) used to assess the robustness of the causal estimate of oily fish intake on PhenoAge.

Variable	Method	# SNP	Beta (95% CI)	P-value
**GrimAge**				
Fish Oil Supplement	MR Egger	4	-1.51(-32.85 to 29.82)	9.33e-01
Fish Oil Supplement	Weighted median	4	-4.51(-10.15 to 1.13)	1.17e-01
Fish Oil Supplement	IVW	4	-5.21(-10.13 to -0.30)	3.77e-02*
Fish Oil Supplement	Simple mode	4	-3.88(-11.58 to 3.82)	3.96e-01
Fish Oil Supplement	Weighted mode	4	-3.80(-11.38 to 3.78)	3.98e-01

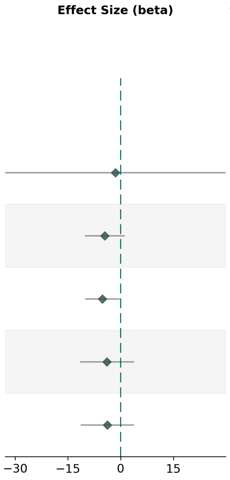

Fig. 3S All 5 MR Methods for Fish Oil vs GrimAge Forest plot showing additional MR methods of simple mode, weighted mode, and weighted median for this statistically significant exposure outcome pair.

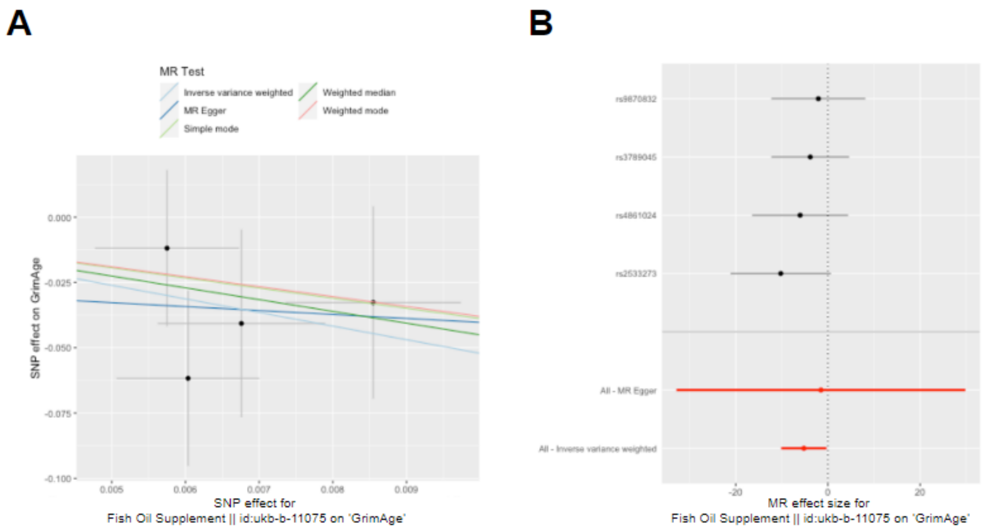

Fig. 4S Sensitivity Analysis of Fish Oil Intake on GrimAge. Scatter plot of IV effects on 5 MR methods (A) and LOO analysis (B) used to evaluate the robustness of the causal estimate of fish oil intake on GrimAge

Variable	Method	# SNP	Beta (95% CI)	P-value
**Tryglycerides**				
Fish Oil Supplement	MR Egger	4	-0.09(-5.16 to 4.99)	9.76e-01
Fish Oil Supplement	Weighted median	4	-0.93(-1.85 to -0.01)	4.85e-02*
Fish Oil Supplement	IVW	4	-1.15(-1.93 to -0.37)	4.00e-03*
Fish Oil Supplement	Simple mode	4	-0.88(-2.19 to 0.44)	2.82e-01
Fish Oil Supplement	Weighted mode	4	-0.82(-2.05 to 0.41)	2.83e-01

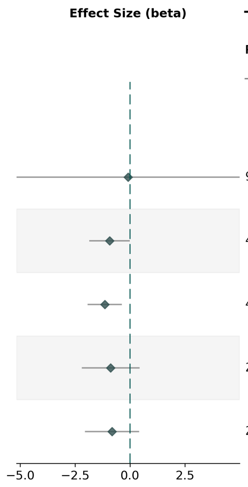

Fig. 5S All 5 MR Methods for Fish Oil vs Triglycerides Forest plot showing additional MR methods of simple mode, weighted mode, and weighted median for this statistically significant exposure outcome pair.

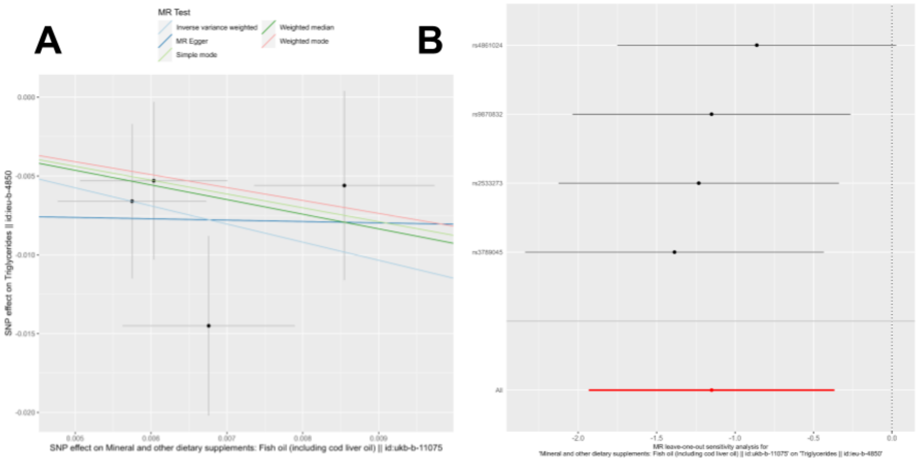

Fig. 6S Fish Oil Triglycerides Sensitivity Scatter plot of IV effects on 5 MR methods (A) and LOO analysis (B) used to assess the robustness of the causal estimate of fish oil supplementation on triglycerides.

Variable	Method	# SNP	Beta (95% CI)	P-value
**Fish Oil Supplement**				
GrimAge	MR Egger	4	-0.09(-0.18 to -0.01)	1.71e-01
GrimAge	IVW	4	-0.00(-0.01 to 0.01)	7.99e-01
Hannum	MR Egger	9	-0.01(-0.02 to 0.01)	2.53e-01
Hannum	IVW	9	-0.00(-0.00 to 0.00)	7.90e-01
IEAA	MR Egger	24	-0.00(-0.01 to 0.00)	4.08e-01
IEAA	IVW	24	-0.00(-0.00 to 0.00)	9.29e-01
PhenoAge	MR Egger	10	-0.00(-0.01 to 0.01)	9.76e-01
PhenoAge	IVW	10	-0.00(-0.00 to 0.00)	8.82e-01

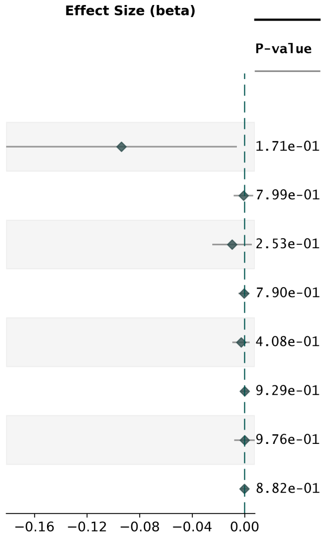

Fig. 7S Reverse MR of Epigenetic Age Measures vs Fish Oil Intake. Beta coefficients and p-values used to represent the potential impact of four epigenetic age measures on fish oil intake. No significant associations were observed.

Variable	Method	# SNP	Beta (95% CI)	P-value
**Oily Fish Inktake**				
GrimAge	MR Egger	4	-0.09(-0.26 to 0.09)	4.27e-01
GrimAge	IVW	4	-0.00(-0.02 to 0.01)	3.75e-01
Hannum	MR Egger	9	0.03(-0.03 to 0.09)	3.73e-01
Hannum	IVW	9	0.00(-0.01 to 0.02)	8.52e-01
IEAA	MR Egger	24	0.00(-0.01 to 0.01)	9.04e-01
IEAA	IVW	24	0.00(-0.00 to 0.01)	7.69e-01
PhenoAge	MR Egger	10	0.01(-0.00 to 0.03)	1.92e-01
PhenoAge	IVW	10	0.00(-0.01 to 0.01)	8.83e-01

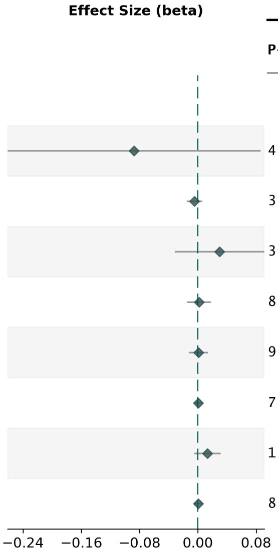

Fig. 8S Reverse MR of Epigenetic Age Measures vs Oily Fish Intake. Beta coefficients and p-values used to depict the potential influence of four epigenetic age measures on oily fish intake. No significant associations were observed.

Variable	Method	# SNP	Beta (95% CI)	P-value
**Fish Oil Supplement**				
C-Reactive Protein Level	MR Egger	53	0.00(-0.00 to 0.01)	2.86e-01
C-Reactive Protein Level	IVW	53	0.00(-0.00 to 0.01)	1.49e-01
C-Reactive Protein Level	MR Egger	28	0.01(-0.01 to 0.03)	2.77e-01
C-Reactive Protein Level	IVW	28	0.00(-0.01 to 0.01)	8.54e-01
HDL Cholesterol	MR Egger	32	-0.00(-0.01 to 0.01)	8.38e-01
HDL Cholesterol	IVW	32	0.00(-0.01 to 0.01)	7.51e-01
LDL Cholesterol	MR Egger	41	0.01(-0.00 to 0.01)	9.62e-02
LDL Cholesterol	IVW	41	0.00(-0.00 to 0.01)	1.27e-01
Tryglycerides	MR Egger	37	0.00(-0.02 to 0.02)	7.19e-01
Tryglycerides	IVW	37	0.01(-0.00 to 0.02)	2.87e-01

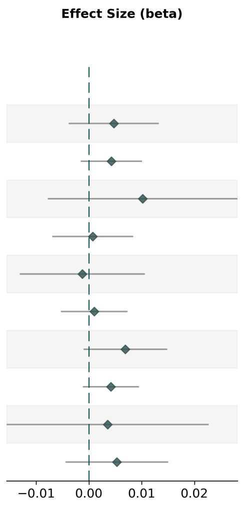

Fig. 9S Reverse MR of Blood Biomarkers vs Fish Oil Supplement. Beta coefficients and p-values illustrating the influence of blood biomarkers on fish oil supplementation. No significant associations found.

Variable	Method	# SNP	Beta (95% CI)	P-value
**Oily Fish Inktake**				
C-Reactive Protein Level	MR Egger	53	-0.02(-0.06 to 0.01)	1.41e-01
C-Reactive Protein Level	IVW	53	-0.01(-0.03 to 0.02)	6.36e-01
HDL Cholesterol	MR Egger	32	0.01(-0.02 to 0.04)	7.26e-01
HDL Cholesterol	IVW	32	-0.00(-0.02 to 0.01)	6.46e-01
Tryglycerides	MR Egger	37	0.06(0.01 to 0.11)	2.76e-02*
Tryglycerides	IVW	37	0.04(0.02 to 0.07)	7.16e-04*

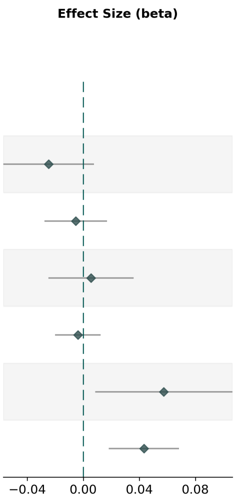

Fig. 10S Reverse MR of Blood Biomarkers vs Oily Fish Intake. Beta coefficients and p-values demonstrating the influence of blood biomarkers on oily fish intake. Significant reverse MR associations ($p < 0.05$) were found for LDL cholesterol and triglycerides on oily fish intake.
